# CRISPR/Cas9 and AAV mediated insertion of β2 microglobulin-HLA-G fusion gene protects mesenchymal stromal cells from allogeneic rejection and potentiates the use for off-the-shelf cell therapy

**DOI:** 10.1016/j.reth.2022.09.009

**Published:** 2022-10-14

**Authors:** Sohsuke Meshitsuka, Ryo Ninomiya, Tokiko Nagamura-Inoue, Takashi Okada, Muneyoshi Futami, Arinobu Tojo

**Affiliations:** a Department of Molecular Therapy, Advanced Clinical Research Center, The Institute of Medical Science, The University of Tokyo, Tokyo 108-8639, Japan; b Keijinkai Medical Corporation, Tokyo 160-0008, Japan; c Daiwa Pharmaceutical Co, Ltd., Tokyo 154-0024, Japan; d Department of Cell Processing and Transfusion/Laboratory Medicine, IMSUT Hospital, The Institute of Medical Science, The University of Tokyo, Tokyo 108-8639, Japan; e Division of Molecular and Medical Genetics, The Institute of Medical Science, The University of Tokyo, Tokyo 108-8639, Japan; f Project Division of Innovative Diagnostics Technology Platform, The Institute of Medical Science, The University of Tokyo, Tokyo 108-8639, Japan; g Institute of Innovation Advancement, Tokyo Medical and Dental University, 113-8510, Japan

**Keywords:** UC-MSCs, AAV, CRISPR/Cas9, HLA-G, Allogenic rejection, AAV, adeno-associated virus, FASL, FAS ligand, GVHD, graft versus host disease, HLA, human leukocyte antigen, HR, homologous recombination, HSC, hematopoietic stem cells, ITR, inverted terminal repeats, KIR, killer-cell immunoglobulin-like receptors, LILR, leukocyte immunoglobulin-like receptors, MLR, mixed lymphocyte reaction, MSC, mesenchymal stromal cells, PBMC, peripheral blood mononuclear cells, PS, penicillin–streptomycin, SD, standard deviation

## Abstract

**Introduction:**

Mesenchymal stromal cells (MSCs) hold the potential for application as cellular therapy products; however, there are many problems that need to be addressed before the use in clinical settings, these include the heterogeneity of MSCs, scalability in MSC production, timing and techniques for MSC administration, and engraftment efficiency and persistency of administered MSCs. In this study, problems regarding immune rejection caused by *human leukocyte antigen* (HLA) mismatches were addressed.

**Methods:**

Umbilical cord-derived MSCs (UC-MSCs) were gene-edited to avoid allogeneic immunity. The HLA class I expression was abrogated by the knock-out of the beta-2-microglobulin (B2M) gene; instead, the B2M-HLA-G fusion gene was knocked-in using the CRISPR/Cas9 system in combination with adeno-associated virus (AAV).

**Results:**

Cell surface markers on gene-edited UC-MSCs were not different from those on primary UC-MSCs. The gene-edited UC-MSCs also retained the potential to differentiate into adipocytes, osteoblasts, and chondrocytes. B2M gene knock-out alone protected cells from allogeneic T cell immune responses but were vulnerable to NK cells. B2M gene knock-out in combination with B2M-HLA-G knock-in protected cells from both T cells and NK cells. The B2M-HLA-G knock-in MSCs retained a good immunosuppressive ability and the addition of these cells into the mixing lymphocyte reaction showed a significant inhibition of T cell proliferation.

**Conclusions:**

The results of this study demonstrated the possibility that the CRISPR/Cas9 system combined with AAV can be used to effectively disrupt/introduce any gene into UC-MSCs. Our findings suggest that the gene-edited cell line produced here using this method may have a higher ability to escape the cytotoxic activity of immune cells than primary cells, thereby being more advantageous for long-term graft survival.

## Highlights

• The CRISPR/Cas9 system with AAV1/2 could knock in/out efficiently in UC-MSCs.
• B2M-HLA-G fusion gene was successfully introduced and expressed to the cell surface.
• Gene edited UC-MSCs were capable of evading allogeneic immune responses.

## 1 Introduction

In recent years, mesenchymal stromal cells (MSCs) have garnered attention as a tool for regenerative medicine. MSCs were first found in human bone marrow in 1999 [1], and subsequently in various other tissues, such as dental pulp, umbilical cord, and adipose tissues. MSCs can differentiate into cells of ectodermal and endodermal lineages, such as neurons and hepatocytes, respectively, as well as cells of the mesenchymal lineage. With its minimal invasiveness, the use of MSCs in humans has little concern to provoke ethical issues. Therefore, MSCs are thought to hold a high potential as a tool for regenerative medicine. MSCs are also known to be capable of immunosuppression. MSCs produce transforming growth factor-β (TGF-β), prostaglandin E2, indoleamine 2,3-dioxygenase (IDO), and other substances that have suppressive effects on the growth of immune cells such as dendritic cells, T cells, and NK cells [2]. MSCs express FAS ligand (FASL) that have also been reported to induce T cell apoptosis [3]. Based on these immunosuppressive effects, MSCs have become in practical use as a cellular therapy for patients with graft versus host disease (GVHD) and Crohn's disease who require the regulation of excessive immune reactions. For GVHD, allogeneic bone marrow-derived MSCs were reported to have an immunosuppressive effect against steroid-refractory GVHD in 2004 [4], and they have been put into practical use and made commercially available as TEMCELL® HS Injection by JCR Pharmaceuticals (Japan). However, the complete response rate was only approximately 50% in patients who received MSCs for treatment of severe acute GVHD [5]. Other points that require improvement include the dosing regimen (twice weekly for 2–4 weeks), efficacy, and persistency. With such a high expectation, investigations on MSCs as a tool for therapeutic management of diseases have recently increased in number progressively; however, the path to implement the clinical application of MSCs is not straightforward. According to the ClinicalTrials.gov database (https://clinicaltrials.gov/), there are 1014 completed or ongoing clinical studies using MSCs as of July 2021, but not a single product has been approved by the FDA, highlighting the challenges that exist before clinical application [6]. The various specific challenges have been identified previously, including the heterogeneity of MSCs [7], scalability in MSC production [8], timing and techniques for MSC administration [9], and engraftment efficiency [10] and persistency [11] of administered MSCs. In this study, immune rejection due to *human leukocyte antigen* (HLA) mismatches was considered as a major issue. Specifically, even in allogeneic MSC transplantation, MSCs with mismatched HLA are eliminated by the host immune system. A possible approach to improve the effectiveness is transplantation between HLA-matched donor–recipient combinations; however, this approach requires cell bank management based on HLA types and imposes heavy economic and social burdens. Thus, in this study, we aimed for the so-called universalization of MSCs to solve the problem of HLA mismatches. We predict that this approach will allow for immediate use after disease onset because of the off-the-shelf availability, sustained efficacy owing to long-term retention in the recipient after transplantation, or facilitated tissue repair because of improved persistency.

**Fig. 1:**
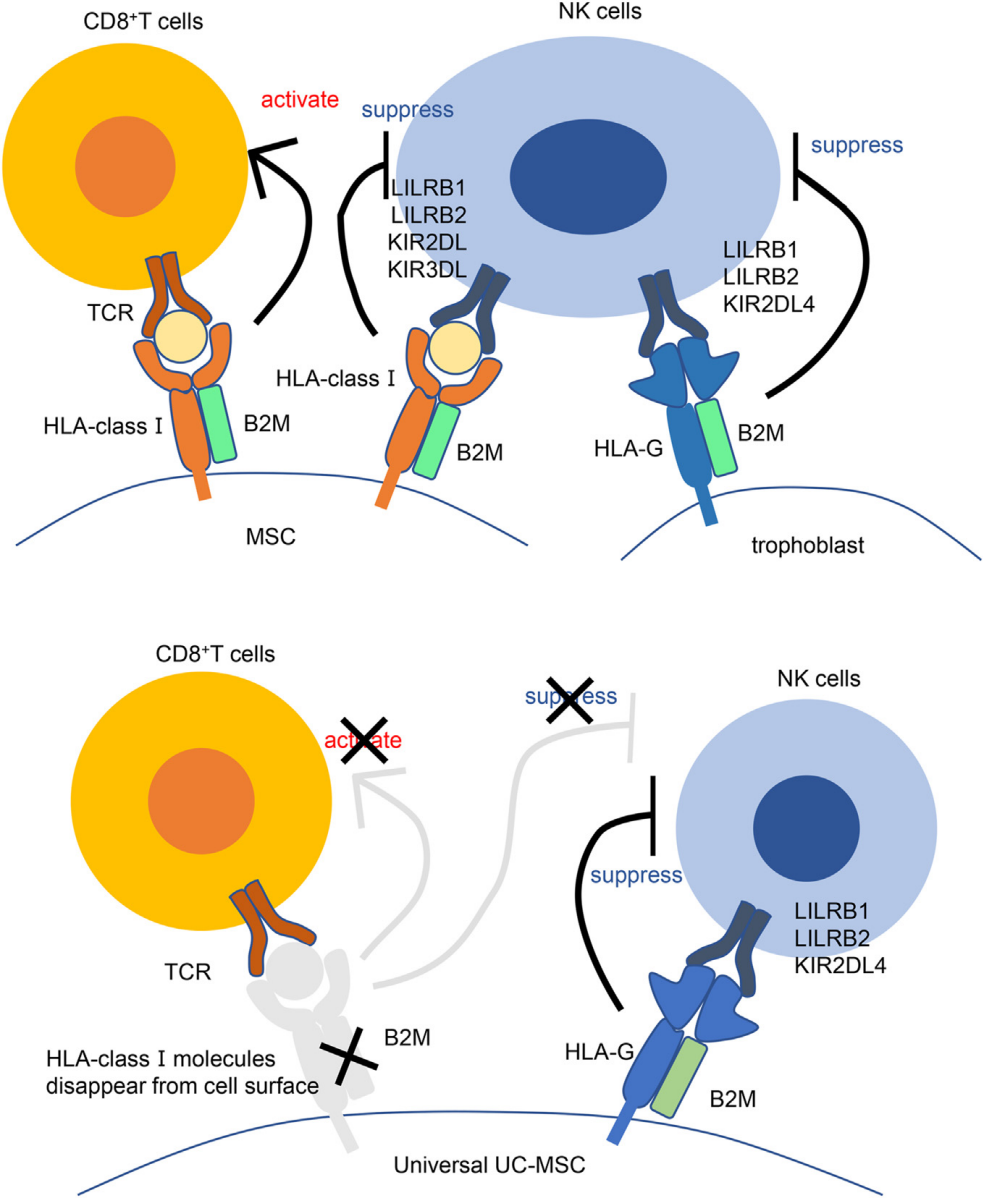
**Universal UC-MSCs escape attacks by cytotoxic T lymphocytes and NK cells by the action of the B2M-HLA-G fusion protein expressed.** The schematic diagram of the universal UC-MSCs that acquired immune tolerance as intended in this study is shown. The B2M knock-out results in the disappearance of HLA-class I from the cell surface. Therefore, the cells are no longer recognized by receptors on CD8-positive T cells (TCRs), acquiring the ability to escape T cell-mediated immunity. In addition, HLA-G expressed as a fusion protein with B2M supplements the ability of HLA-class I to suppress NK cells.

To achieve the universalization of MSCs while utilizing the advantage of their immunosuppressive ability, we knocked out HLA-class I which causes immune rejection in allogenic MSC transplantation, then replaced it with HLA-G, which has only a scarce number of gene polymorphism. We used umbilical cord MSCs (UC-MSCs) as the resource owing to the fact that they have excellent characteristics as a therapeutic resource, such as non-invasiveness during collection (they are obtained from umbilical cord tissues, which are otherwise discarded after delivery) and a high MSC content. HLAs are cell surface antigens playing a central role for human immune responses, and in particular, HLA-class I molecules initiate immune responses in transplantation by presenting antigens to CD8-positive T cells. It is known that HLA-class I molecules can be expressed on the cell surface after binding to B2M molecules intracellularly [12], and the cell surface expression of HLA-class I molecules, such as HLA-A, -B, and -C, are known to require the binding with B2M. B2M gene knock-out in cell lines, iPS cells, and primary human MSCs abrogates HLA class I expression [13]. One may expect that if this is used to eliminate HLA-class I molecules from the MSC surface, it may allow MSCs to escape recognition by TCR on recipient T cells. However, once HLA-class I molecules are eliminated, NK cell-inhibiting signals via certain types of killer-cell immunoglobulin-like receptors (KIRs; such as KIR2DL and KIR3DL) and leukocyte immunoglobulin-like receptors (LILRs; such as LILRB1 and LILRB2) are lost, and MSCs are then attacked by NK cells [14]. Thus, transplantation of cells expressing no HLA-class I molecules on their surface is likely to result in engraftment failure due to NK cell activation. HLA-G is one of HLA-class Ib molecules, and the HLA-G gene is located on chromosome 6 (6p21). Compared with other HLA-class I molecules, HLA-G is characterized by a distinct tissue distribution pattern and a smaller number of gene polymorphisms in the coding region and is known to contribute to maternal–fetal immune tolerance in particular. HLA-G suppresses the NK cytotoxic activity by being recognized as a ligand by inhibitory receptors LILRB1, LILRB2, and KIR2DL4, which are expressed on the surface of NK cells [15,16]. Taken together, to avoid recognition by T cells and cytotoxicity of NK cells, we designed UC-MSCs in which B2M gene is knocked out to eliminate cell surface HLA-class molecules for escaping from TCR recognition, and in its place the B2M-HLA-G fusion gene is knocked in to inhibit cytotoxicity of NK cells (Fig. 1).

There are seven known isoforms of HLA-G (G1–G7). Both LILRB1 and LILRB2 are receptors for HLA-G, and their intracellular domains transmit inhibitory signals. LILRB1 recognizes the HLA-G α3 domain and B2M as a unit. Thus, LILRB1 does not bind to HLA-G without B2M [17,18]. However, LILRB2 recognizes the HLA-G α3 domain and can consequently bind to HLA-G with or without B2M [18,19]. On the other hand, HLA-G without B2M has been reported to bind to KIR2DL4 receptors, inducing increases in interferon γ (IFNγ) production [20] and cytotoxicity [21]. Thus, in this study, we decided to introduce the HLA-G gene linked with B2M using (G_4_S)_3_ linker to suppress NK cell activity by mimicking the HLA-G1 isoform.

Whether or not HLA-G-positive and HLA-class I-negative MSCs can actually escape immune responses and avoid cytotoxicity of T cells and NK cells in an allogeneic transplantation system has not been demonstrated thus far. As to HLA-G gene transduction methods, adequate direct comparisons have not been made between cells produced through the conventional lentivirus-based method (i.e., random integration) and cells prepared with specific editing of the target genes. In the present study, we produced UC-MSCs in which B2M was knocked out to eliminate cell surface HLA-class I molecules and B2M-HLA-G fusion gene was introduced with different methods. One was with a lentivirus vector, and the other was with a CRISPR/Cas9 system with adeno-associated virus (AAV) as the DNA donor, and we compared their efficacies.

The universalized UC-MSCs are considered more convenient in various aspects, such as the off-the-shelf availability, no requirement for cell banks, reduced production cost, and the increased value as a carrier. In addition, the cells resistant to elimination would persist in the body for a long term, and we thought that UC-MSCs may hold the potential for applications requiring long-term effect or tissue repair. Furthermore, the direct action of expressed HLA-G may increase the immunomodulatory ability, providing an additional benefit in the treatment of certain diseases. Therefore, UC-MSCs prepared here may solve the issues currently experienced in cell therapy.

## 2 Material and methods

### 2.1 Ethics

This study was in consistent with the relevant provisions of the 1964 Helsinki Declaration. This study was approved by the Institutional Review Board at the Institute of Medical Science of the University of Tokyo (approval no. 29–28, A0722/2021-108; Tokyo, Japan), including obtaining UC-MSCs and cord blood, peripheral blood mononuclear cells (PBMCs) from healthy donors. Primary cells derived from the patients who signed informed consent form were used.

### 2.2 Cell culture

UC-MSCs were provided by Human umbilical cord blood and cord bank (IMSUT CORD, Tokyo, Japan). UC-MSCs were aseptically cultured in minimum essential medium α (MEMα, FUJIFILM Wako Pure Chemical Corporation) supplemented with 10% heat inactivated fetal bovine serum (FBS, GE healthcare) and 1% penicillin–streptomycin (PS, FUJIFILM Wako Pure Chemical Corporation) in a 5% CO_2_ incubator at 37 °C, and subcultured every time the cells reached 70%–90% confluency. When subculturing, the medium was removed from the culture container via aspiration, and the cells were washed once with Dulbecco's phosphate-buffered saline (D-PBS, Nacalai Tesque, Inc.). Cells were detached using TrypLE Express Enzyme (1X) and phenol red (Thermo Fisher Scientific), and collected and washed with fresh medium. An arbitrary number of cells were seeded in a new culture container. PMDC05 cells were provided by Dr. Narita at the Faculty of Medicine, Niigata University [22]. PMDC05 cells were aseptically cultured using RPMI 1640 medium (Nacalai Tesque) supplemented with 10% FBS and 1% PS in a 5% CO_2_ incubator at 37 °C, and subcultured every 3–4 days. KHYG-1 cells were purchased from the Health Science Research Resource Bank (HSRRB, Japan). KHYG-1 cells were aseptically cultured using RPMI 1640 medium supplemented with 10% FBS, 1% PS, and 20 ng/mL recombinant human IL-2 (PEPROTECH) in a 5% CO_2_ incubator at 37 °C, and subcultured every 3–4 days. Lenti-X293T cells (Clontech; Takara Bio USA) were cultured in DMEM (FUJIFILM Wako Pure Chemical Corporation) with 10% FBS and 1% PS, at 37 °C with 5% CO_2_.

### 2.3 Lentiviral production and transduction

Lentiviral plasmid (CSII-EF-MCS and CSII-CMV-MCS) was obtained from RIKEN Bioresource Center. Lentiviral plasmid (CSII-EF-fLuc-2A-EGFP) was produced as described previously [23]. Lentiviral vector particles were produced by cotransfection of Lenti-X293T cells with a transfer plasmid, and packaging plasmids pMDLg/p.RRE, pRSV-rev, and pMD.G at 37 °C with 5% CO_2_ for 2 days. The lentiviral particles (LV- EF-fLuc-2A-EGFP and LV-CMV-B2M-G4S-HLA-G) were titrated by the transduction of HeLa cells, and stored at −80 °C until use. B2M(−) MSCs were transduced with the LV-CMV-B2M-G4S-HLA-G at a multiplicity of infection of 100. For the luciferase reporter assay, each UC-MSCs was transduced with the LV-EF-fLuc-2A-EGFP lentiviral vector at a multiplicity of infection of 20. Seven days after transduction, target cells were harvested using FCM.

### 2.4 Guide RNA synthesis

Guide RNA used in gene knock-out/knock-in with the CRISPR/Cas9 system was synthesized as follows: To knock out B2M by gene editing, an 18-bp target sequence found in the human B2M gene sequence and predicted to have low homology on the other genes was determined by CRISPRdirect (http://crispr.dbcls.jp/). An oligo DNA in which the T7 promoter region and the tracrRNA sequence containing Cas9 protein binding site were added to this target sequence was designed according to the package insert of Guide-it sgRNA In Vitro Transcription Kit (Takara Bio). The designed sequence was artificially synthesized as a primer (FASMAC), and the product was used as the template to synthesize a single gRNA (sgRNA) according to the kit. The target sequence and PAM sequence are shown in Supplementary Table 1.

### 2.5 AAV vector construction and AAV packaging

**Fig. 2:**
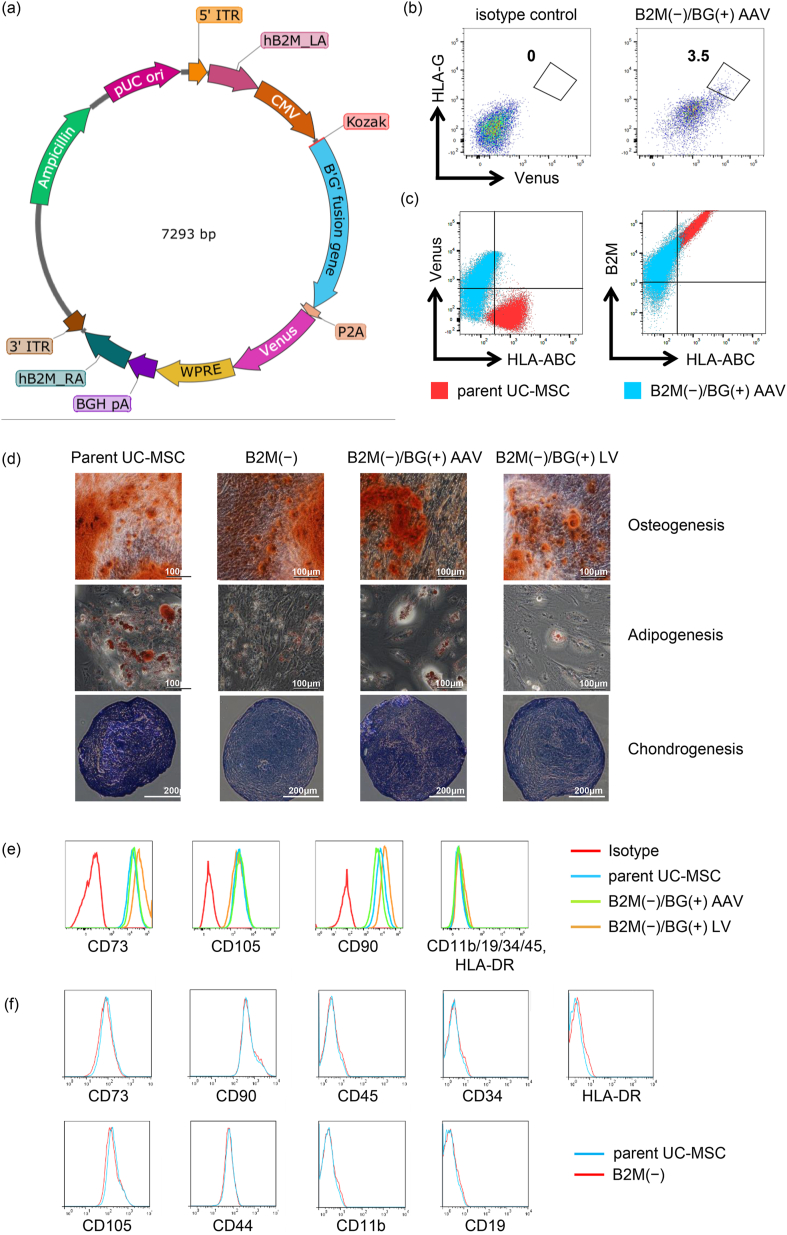
**UC-MSCs in which a gene encoding the B2M-HLA-G fusion protein was introduced at the B2M gene locus by CRISPR/Cas9-AAV retained the characteristics as MSCs.** a) The schematic diagram of the plasmid sequence packaged into AAV. The plasmid sequence contains the codon optimized B2M-G4S-HLA-G sequence under the control of CMV promoter and was designed to allow for continuous transcription of the downstream Venus sequence by the P2A sequence. b) Four days after gene transfer was performed, the presence of desired cells (B2M(−)/BG(+) AAV) was confirmed via FCM. c) The expression extents of HLA-ABC and B2M on the surface of B2M(−)/BG(+) AAV and the Venus fluorescence intensity were analyzed by FCM. d) Osteogenesis, adipogenesis, and chondrogenesis differentiation potentials of various UC-MSCs were confirmed. The cells cultured using differentiation induction medium were stained with alizarin red, Oil red O, and toluidine blue. e,f) Cell surface markers of various UC-MSCs were analyzed by FCM. Histograms of FCM results for isotype (red in e), parent UC-MSC (light blue), B2M(−)/BG(+) AAV (yellowish green), B2M(−)/BG(+) LV (orange), B2M(−) (red in f) are shown. The results of CD73, CD90, CD105, CD11b, CD19, CD34, CD45, and HLA-DR, which are the MSC criteria, are shown.

The sequence of AAV vector for knock-in was constructed as follows: We designed the B′G′ fusion gene, in which the HLA-G and B2M genes were fused via (G_4_S)_3_ linker repeating GGGGS for three times, and the resulting gene for B2M-HLA-G fusion protein underwent codon optimization to avoid unwanted homologous recombination (HR), while keeping its amino acid sequence intact. A Venus fluorescent protein gene was added at the downstream of the B′G′ fusion gene via P2A sequence, and put it under the CMV promoter. Homologous regions from B2M were added for HR at the upstream and downstream sides (400 bp each) of the designed sequence. The plasmid containing the resulting sequence flanked by two inverted terminal repeats (ITRs) for incorporation into an AAV vector was artificially synthesized (Fig. 2a, VectorBuilder). Based on this plasmid, AAV1/2 with serotype 1 capsid proteins and serotype 2 ITRs was produced and used for knock-in experiments.

### 2.6 Knock-in/knock-out experiment of DNA vector via electroporation

Gene knock-in/knock-out in UC-MSCs was performed as follows. Cells for the experiment were precultured, and cells in the logarithmic growth phase were prepared. A mixture of 10 μg of Guide-it Recombinant Cas9 protein (Electroporation-Ready, Takara Bio) and 2.5 μg of sgRNA as described in Section 2.4 was let stand to react for 10 min at 23–27 °C. The cells (1.0 × 10^6^) were suspended in 100 μL of culture medium, and 5 μg of plasmid vector was added to the cell suspension. However, the vector was not added when only knock-out was to be performed (e.g., when B2M(−) was produced). The mixture of sgRNA and Cas9 was added to this cell suspension, and electroporation was conducted promptly. The CUY21 Pro-Vitro In Vitro electroporator (NEPA GENE) was used for gene transfer, and the setting conditions of the electroporator were P-, (150 V 10 ms) × 1 cycle, (20 V 50 ms) × 10 cycles, and off time: 50 ms. After electroporation, cells were suspended in 10 mL of fresh medium and seeded in a 10-cm dish. The cells grown as described in Section 2.1 were used for various experiments.

### 2.7 Gene knock-in experiment using AAV

A mixture of 10 μg of Cas9 protein and 2.5 μg of sgRNA was incubated to react for 10 min at 23–27 °C. The sgRNA-Cas9 mixture was added to a suspension of MSCs (1.0 × 10^6^ cells per 100 μL of MEMα medium), and electroporation was operated promptly. After electroporation, UC-MSCs were suspended in 5 mL of MEMα medium, and >10^11^ genome copies of AAV vector were promptly added to the cell suspension. The cells were seeded in a 10-cm dish and cultured for 16–24 h under usual cell culture conditions. Then, the medium was replaced with fresh MEMα medium at least twice, and culturing was continued. The cells were cultured until a sufficient number of cells were obtained, and then used for various experiments.

### 2.8 Flow cytometry (FCM) analysis

UC-MSCs in this study were analyzed according to a standard flow cytometry protocol. Specifically, the cultured cells were collected, washed with D-PBS containing 0.1% bovine serum albumin (Nacalai Tesque, Inc.), and then stained using proper FCM-grade, fluorescently labeled antibodies for human cells and fluorescently labeled antibodies for isotype control. Dead cells were stained with propidium iodide. Cells were analyzed with a FACSCanto II or FACSCelesta flow cytometer (Becton–Dickinson Biosciences), and were sorted with a FACSAria, SORPAria, FACSAria III (Becton–Dickinson Biosciences), or SH800 cell sorter (SONY Corporation). Data were analyzed with FACSDiva software, SH800 Cell Sorter software, or FlowJo software. The labeled antibodies used for FCM analysis in this study are shown in Supplementary Table 2.

### 2.9 Mixed lymphocyte reaction (MLR)

UC-MSCs precultured were collected and resuspended in MEMα medium at 8.0 × 10^4^ cells/mL. The UC-MSC suspension was dispensed into a 24-well plate (500 μL/well) and then cultured for 2–3 h until the cells adhered to the plate. PMDC05 cells precultured were collected and resuspended in RPMI 1640 medium at 2.0 × 10^5^ cells/mL. This suspension was irradiated with 50-Gy radiation to cease cell proliferation. PBMCs from healthy donors were suspended in D-PBS and fluorescently labeled using CellTrace Far Red Cell Proliferation Kit, for flow cytometry (Thermo Fisher Scientific). The specific procedures conformed with the package insert. As cells stained with the CellTrace reagent can be handled in the same manner as cells stained with CFSE, a staining dye for living cells, and the intensity of reagent-derived fluorescence is halved each time cells undergo division, the proliferation of PBMCs can be traced by analyzing the fluorescence intensity and cell population distribution. Stained PBMCs were resuspended in RPMI 1640 medium at 1.0 × 10^6^ cells/mL. The PBMC suspension was added to the 24-well plate (200 μL/well) in which UC-MSCs had been seeded. The proliferation of PBMCs was evaluated after the addition of the suspension of PMDC05 cells (200 μL/well) as stimulator cells followed by Lymactin-T (final concentration, 0.75 ng/mL; Cell Science & Technology Institute Inc.) as an anti-CD3 antibody for the reaction enhancement (MSC:PMDC05:PBMCs = 1:1:5). In addition, a series of cell mixtures containing phytohemagglutinin-L (PHA-L, ROCHE) at a final concentration of 10 μg/mL were prepared as positive control samples. The 24-well plate was incubated for 4 days before PBMCs in respective wells were collected. The collected PBMCs were stained with fluorescently labeled anti-CD4 and anti-CD8a antibodies, and the proliferation of CD4-positive T cells and CD8-positive T cells was analyzed by FCM.

### 2.10 Killing assay

UC-MSCs co-cultured with KHYG-1 cells or PBMCs were analyzed using xCELLigence Real time cell analysis (RTCA) DP analyzer (Agilent) to measure changes in the dead cell rate of UC-MSCs over time. This equipment can continuously measure the ionic state of culture medium, morphological changes of cells in contact, and cell proliferation, which are recorded as changes in electrical resistance with microelectrodes placed at the bottom of special 16-well plates. This makes it possible to monitor not only the number of cultured cells, but also other pieces of information such as cell morphology and viability observed as changes in the cell adherence state over time. Various UC-MSCs precultured, collected, and resuspended in MEMα medium were seeded in a special 16-well plate (7500 cells/well) and cultured overnight. The next day, a suspension of KHYG-1 cells or PBMCs as effector cells in RPMI-1640 medium supplemented with IL-2 (final concentration, 20 ng/mL) was prepared and added to each well to dispense the required number of effector cells and initiate the reaction. Changes in electrical resistance were measured with the analyzer every 15 min for approximately 80 h. The obtained values were analyzed with the attached RTCA software Pro (Agilent) and divided by effector cell-derived background resistance values to calculate the normalized cell index values. Furthermore, ratios of differences in the normalized cell index between wells with effector cells and wells without effector cells (%cytolysis) were calculated for different UC-MSCs. In this experimental system, normalized cell index values reflect the proliferative states of UC-MSCs, while %cytolysis values reflect dead cell rates of UC-MSCs.

### 2.11 Induction of UC-MSC differentiation

The differentiation potency of various UC-MSCs was investigated using StemPro Differentiation Kits for adipogenesis/chondrogenesis/osteogenesis (Thermo Fisher Scientific). Specific procedures conformed with the package insert of each kit. Briefly, various MSCs precultured were collected from culture containers and seeded in 24-well plates for the adipogenic and osteogenic potency assays and 15 mL tubes for the chondrogenic potency assay. After the medium was replaced with the induction medium, the cells were cultured at 37 °C and 5% CO_2_, and the medium was replaced every 3–4 days. Cells that underwent adipocyte differentiation were detected by Oil red O (FUJIFILM Wako) staining. Cells that underwent chondrocyte differentiation were detected by toluidine blue staining after fixation in 4% formaldehyde. Cells that underwent osteogenic differentiation were detected by alizarin red (FUJIFILM Wako) staining.

### 2.12 Luciferase reporter assay

For the direct cytotoxicity assay, UC-MSCs were co-cultured with PBMCs at an effector/target ratio of (0, 1, 2, 5, 10):1 at 37 °C for 48 h. Cell death was calculated from the decrease in luciferase activity, which was detected using Steady Glo (Promega Corporation). Luciferase luminescence in the samples was evaluated using a Nivo spectrophotometer (PerkinElmer, Inc.).

## 3 Results

### 3.1 Gene knock-in to UC-MSCs using AAV

Generally speaking, knock-out experiments have high success rate, whereas knock-in experiments have low success rate in gene editing using various cells, especially in the cases of long sequences. A recent study using hematopoietic stem cells (HSCs) has shown that the CRISPR/Cas9 system combined with adeno-associated virus (AAV) can be used as a highly efficient knock-in method to insert DNA sequences at target sequences in genomes via the homologous recombination (HR) repair mechanism [24]. We attempted to use this knock-in method in the present study with the assumption that the method was developed using HSCs but would also be applicable to UC-MSCs.

In the knock-in experiment, AAV1/2 was added for infection promptly after double strand breaks were generated in the B2M sequence in the genome of UC-MSCs using CRISPR/Cas9. Through this procedure, we expected to complete both the knock-out of the genomic B2M and the knock-in of the B′G′ fusion gene in a single step. After infection, the cells were cultured for 4 days and analyzed by FCM. The FCM data revealed that approximately 3.5% of UC-MSCs belonged to the HLA-G-positive Venus-positive population (Fig. 1b, Venus is a fluorescence protein used as a transduction marker). This result showed that the combined use of CRISPR/Cas9 and AAV1/2 was an efficient way to produce UC-MSC lines with a gene introduced in a target sequence-specific manner. The collected HLA-G-positive Venus-positive population was cultured, and the cells were designated as B2M(−)/BG(+) AAV. The FCM analysis of the collected B2M(−)/BG(+) AAV revealed that the cells were B2M protein-positive, while HLA-A, B, and C proteins, which belong to HLA-class I, were no longer expressed on the cell surface as expected (Fig. 1c).

As a cell line for observing effects of B2M deletion, genomic B2M was knocked out using the CRISPR/Cas9 system, and the UC-MSCs obtained were designated as B2M(−). Furthermore, using B2M(−) as the parental cell line, the B2M-HLA-G fusion gene was inserted into the genome at multiple random positions using a lentiviral vector (designated as B2M(−)/BG(+) LV) for a comparison with B2M(−)/BG(+) AAV, in which the gene was introduced in a target-specific manner. For the production of B2M(−)/BG(+) LV, we used a lentivirus, which was used previously reported for the successful introduction of foreign genes into MSCs [25].

### 3.2 Examination of differentiation potentials of the genome-edited UC-MSCs

Differentiation induction experiments were performed to confirm whether genome edited UC-MSCs produced in this study retained the differentiation potentials as MSCs (Fig. 1d). Osteoblasts, adipocytes, and chondrocytes were detected by staining with alizarin red, Oil red O, and toluidine blue, respectively. The results confirmed that all edited UC-MSCs differentiated into bone, adipose, and cartilage tissues. Therefore, gene editing performed in this study was found not to impair the differentiation potentials of UC-MSCs.

### 3.3 Examination of cell surface antigen profiles of the transfected UC-MSCs

FCM analysis was performed to confirm if transfected UC-MSCs produced in this study retained the cell surface antigen profile as MSCs. The results revealed that all gene-edited UC-MSCs, alike the primary UC-MSC used as the positive control, were positive for CD73, CD90, and CD105 and negative for CD11b, CD19, CD34, CD45, and HLA-DR (Fig. 1e and f). These findings indicate that gene editing performed in this study did not impair the cell surface antigen profile of UC-MSCs.

### 3.4 Examination of immune responses to transfected UC-MSCs by mixed lymphocyte reaction

Next, the mixed lymphocyte reaction (MLR) was performed to examine whether the immunosuppressive ability of each gene-edited UC-MSCs is maintained or not. The immunosuppressive effects of UC-MSCs on T cells were evaluated by co-culturing each gene-edited UC-MSCs and human PBMCs for 4 days with PMDC05, an antigen-presenting cell line added as stimulator cells, and an anti-CD3 antibody added to enhance the immune responses (Fig. 2a and b). The effects were also examined in a similar experimental system using PHA-L added as a chemical stimulus (Fig. 2c and d). The results obtained in both the PMDC05 and PHA-L stimulation systems showed significantly decreased proliferation rates for both CD4-positive T cells and CD8-positive T cells when co-cultured with any of UC-MSCs, compared with the proliferation rates for cells in the NoMSC series that were not co-cultured with UC-MSCs (p < 0.05). Meanwhile, although PBMCs co-cultured with B2M(−)/BG(+) AAV tended to show a lower proliferation rate compared with PBMCs co-cultured with parent UC-MSCs, the T cell proliferation rates did not differ significantly among each UC-MSCs co-cultured. These findings suggest that gene-edited B2M(−)/BG(+) AAV retains the suppressive effect on T cell proliferation comparable to that of the primary parent UC-MSCs.

### 3.5 Measurement of the reactivity of gene-edited UC-MSCs to NK cells and T cells by the real-time cell analyzer

This study aimed to produce universal UC-MSCs capable of evading the recipient immune system even when they are used in allogeneic transplantation. To investigate how well the cytotoxicity escape ability was retained by the B2M(−)/BG(+) AAV line, we used the killing assay system in which gene-edited UC-MSCs were co-cultured with PBMCs or the NK cell line KHYG-1. The xCelligence real-time cell analysis analyzer was used to monitor over time the cellular states of UC-MSCs (target cells) co-cultured with effector cells, and normalized cell index, which reflect the proliferation degree of UC-MSCs, were determined. Furthermore, %cytolysis which reflects dead cell rates were calculated from the normalized cell indices of each well in which target and effector cells were co-cultured and that of target cells alone.

**Fig. 3:**
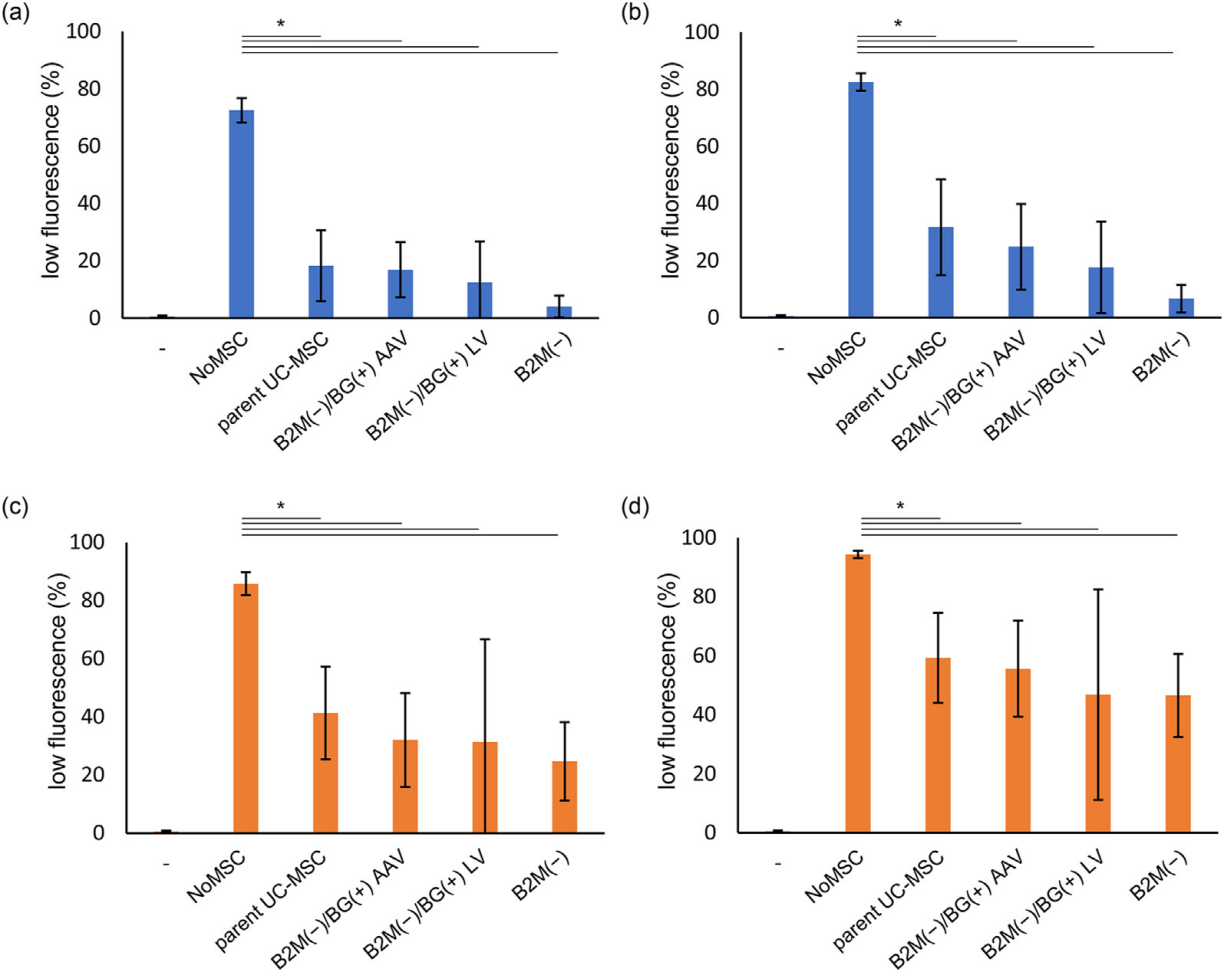
**Gene-edited UC-MSCs suppressed the proliferation of CD4+ T cells and CD8+ T cells.** Various UC-MSCs were precultured and then used for MLR experiments under various conditions. PBMCs collected 4 days after the reaction was initiated were analyzed by FCM (n = 3). a,b) MLR experiments were conducted by adding PMDC05 cells as a stimulator and anti-CD3 antibody as an immunostimulant. a) Percentage of CD4-positive cells that divided at least once. b) Percentage of CD8-positive cells that divided at least once. c,d) PHA-L chemical stimulation induced PBMC division. c) Percentage of CD4-positive cells that divided at least once. d) Percentage of CD8-positive cells that divided at least once. ∗ Represents significant difference (p < 0.05). −; PBMCs only. NoMSC; PBMCs with stimulators. Parent UC-MSC, B2M(−)/BG(+) AAV, B2M(−) or B2M(−)/BG(+) LV; PBMCs with stimulators and indicated UC-MSCs, respectively. Data were expressed as mean ± SD.

%Cytolysis of each UC-MSCs at 10 h after KHYG-1 were seeded as effector cells are shown in Fig. 3a. The median %cytolysis was 64.4 for parent UC-MSC, 69.6 for B2M(−)/BG(+) AAV, 100 for B2M(−), and 44.3 for B2M(−)/BG(+) LV. %Cytolysis of parent UC-MSC, B2M(−)/BG(+) AAV, and B2M(−)/BG(+) LV were significantly lower than that of B2M(−) (p < 0.005). In particular, the %cytolysis of B2M(−)/BG(+) LV was significantly lower than that of B2M(−)/BG(+) AAV, suggesting that B2M(−)/BG(+) LV might have the highest activity to escape the cytotoxicity of KHYG-1 (p < 0.05). Meanwhile, no significant differences were observed between parent UC-MSC and B2M(−)/BG(+) AAV or B2M(−)/BG(+) LV (see Fig. 3).

To further analyze the immune escape ability of gene-edited UC-MSCs, the killing assay was performed using PBMCs as effector cells. %Cytolysis at 30, 50, and 70 h after PBMCs were seeded are shown in Fig. 3b–d. The median %Cytolysis at 30, 50, and 70 h after seeding were: 60.14, 62.75, and 76.79, respectively, for parent UC-MSC; 52.28, 59.24, and 63.91, respectively, for B2M(−)/BG(+) AAV; 66.35, 76.11, and 83.88, respectively, for B2M(−); and 68.82, 77.59, and 86.83, respectively, for B2M(−)/BG(+) LV. While the significance of differences between each UC-MSCs changed over time, in general, the %Cytolysis of B2M(−) and B2M(−)/BG(+) LV were similarly high, and the %Cytolysis of B2M(−)/BG(+) AAV was the lowest. The %Cytolysis of parent UC-MSC was the intermediate between the values of B2M(−) or B2M(−)/BG(+) LV and B2M(−)/BG(+) AAV, but the %Cytolysis of parent UC-MSC increased after 70 h and became significantly higher than that of B2M(−)/BG(+) AAV (p < 0.05). These results suggest that B2M(−)/BG(+) AAV has a high ability to escape the cytotoxicity of PBMCs and remains effective for a long period of time.

### 3.6 Measurement of the reactivity of gene-edited UC-MSCs to NK cells and T cells by the luciferase assay

The luciferase assay was performed to further characterize the immune evasion ability of B2M(−)/BG(+) AAV. A plasmid carrying fLuc-EGFP was transfected into parent UC-MSC, B2M(−)/BG(+) AAV, B2M(−), and B2M(−)/BG(+) LV, to produce UC-MSCs transiently expressing the firefly luciferase (fLuc) protein.

**Fig. 4:**
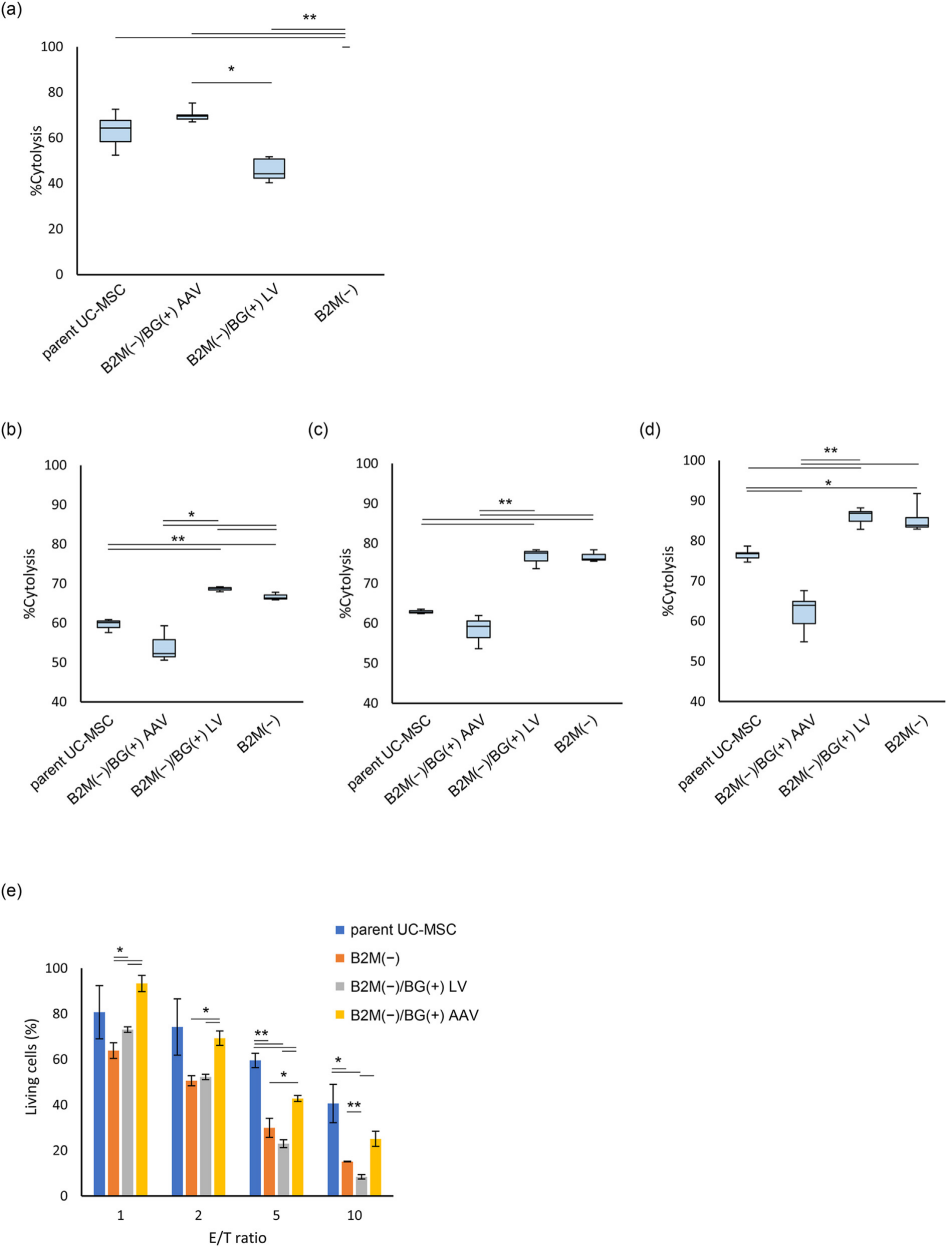
**B2M(−)/BG(+) AAV had a high cytotoxicity escape ability and was advantageous for long-term survival.** a–d) %Cytolysis values were obtained by the killing assay. Box-and-whisker plots for various UC-MSCs co-cultured with KHYG-1 at an E/T ratio of 1.25 for 10 h are shown (a). Box-and-whisker plots for various UC-MSCs co-cultured with PBMCs at an E/T ratio of 2.5 for 30 h (b), 50 h (c), and 70 h (d) are shown. e) The luciferase activity levels of various UC-MSCs transiently expressing fLuc-EGFP were measured after they were co-cultured with PBMCs at E/T ratios of 0, 1, 2, 5, and 10 for 48 h. Ratios of fluorescence intensities to the intensity at an E/T ratio of 0 were calculated and are shown in a bar graph as viability. ∗ and ∗∗ represents significant difference (p < 0.05) and extremely significant difference (p < 0.01), respectively.

These UC-MSCs as target cells were co-cultured with PBMCs as effector cells, and the percentage of MSC viability were estimated from the luciferase activity levels measured after 48 h (Fig. 4e). To test the impact of the effector/target ratio to the viability as well, experiments were conducted with different E/T ratios of 1, 2, 5, and 10. The results showed that the viability of B2M(−)/BG(+) AAV were higher than those of B2M(−) and B2M(−)/BG(+) LV at all E/T ratios tested, suggesting that B2M(−)/BG(+) AAV has a higher ability to escape the cytotoxicity from PBMCs than the other two. In the comparison with parent UC-MSC, however, percentage of living cells in B2M(−)/BG(+) AAV were comparable or higher at E/T ratios of 1 and 2, but were conversely lower at E/T ratios of 5 and 10.

## 4 Discussion

In this study, we demonstrated that the combined use of the CRISPR/Cas9 system and AAV was effective for knock-in of the codon optimized B2M-HLA-G fusion gene specifically at the target B2M gene locus and that the fusion protein produced from the introduced gene was expressed on the cell surface. HLA-class I molecules bind to TCR on T cells and KIR and LILR on NK cells to control immune responses in allogeneic transplantation. HLA-A-, B-, and C-negative cells produced in this study are considered to evade the cytotoxicity of CD8-positive T cells as they are not recognized by TCR. However, HLA-A-, B-, and C-negative cells provoke NK cell-mediated immune responses, as they do not provide inhibitory signals to KIR2DL, KIR3DL, LILRB1, and LILRB2 on NK cells. Thus, in this study, we attempted to establish gene-edited cells capable of evading immune responses mediated by not only T cells but also NK cells. It has been reported that the cells expressing HLA-G escape attacks from NK cells via the LILRB1, LILRB2, and KIR2DL4 receptors. Therefore, we expected that UC-MSCs of which the B2M gene was selectively knocked out and edited to eliminate cell surface HLA-A, B, and C and express the B2M-HLA-G fusion protein could evade the cytotoxicity of both T cells and NK cells and be advantageous for successful engraftment in allogeneic transplantation. Gornalusse et al. have reported that embryonic stem cells expressing only HLA-E can escape allogeneic responses of NK cells and T cells [26]; however, effects of HLA-G, which is also classified as HLA-class Ib, on immunomodulation have not been fully elucidated. The differentiation potency of UC-MSCs is known to decrease as they undergo more cell divisions. This is an obstacle to clinically applying MSCs as cellular therapies and for other purposes, and the development of an efficient gene knock-in method is awaited. In this study, to solve this problem, we examined an efficient target-specific gene knock-in method in UC-MSCs and found that HR-based gene editing with the CRISPR/Cas9 system and AAV1/2 in combination was highly efficient.

In 2006, Dominici et al. have proposed the minimal criteria to define human MSCs as the Mesenchymal and Tissue Stem Cell Committee of the International Society for Cellular Therapy [27]. First, MSCs must be plastic-adherent when maintained in standard culture conditions. Second, MSCs must differentiate to osteoblasts, adipocytes and chondroblasts *in vitro*. Third, MSCs should be positive for CD105, CD73 and CD90, and negative for CD45, CD34, CD14/CD11b, CD79a/CD19, and HLA-DR cell surface markers. Since all of gene-edited UC-MSCs in this study were confirmed to meet the definition, it was suggested that knock-in/knock-out processes, including CRISPR/Cas9 system with AAV, do not affect the characteristics of UC-MSCs.

UC-MSCs have been reported to exert inhibitory effects on T cells by secreting humoral factors, such as TGFβ, IL-10, and IDO [28], and we conducted MLR assays to see if B2M(−)/BG(+) AAV and B2M(−)/BG(+) LV with the introduced B2M-HLA-G fusion gene retain similar immunosuppressive potentials. The immunosuppressive effects of bone marrow-derived MSCs against steroid-refractory GVHD have been reported in 2004 [5]. Our results in this study showed that MSCs suppressed T cell proliferation, being consistent with previous studies showing the immunosuppressive effects of MSCs. The suppression of T cell division by UC-MSCs has also been reported [29], being in agreement with the results of the present study. B2M(−)/BG(+) AAV, a cell line that underwent AAV-mediated gene transfer, suppressed T cell division more effectively than parent UC-MSC, albeit the difference was not of significance.

**Fig. 5:**
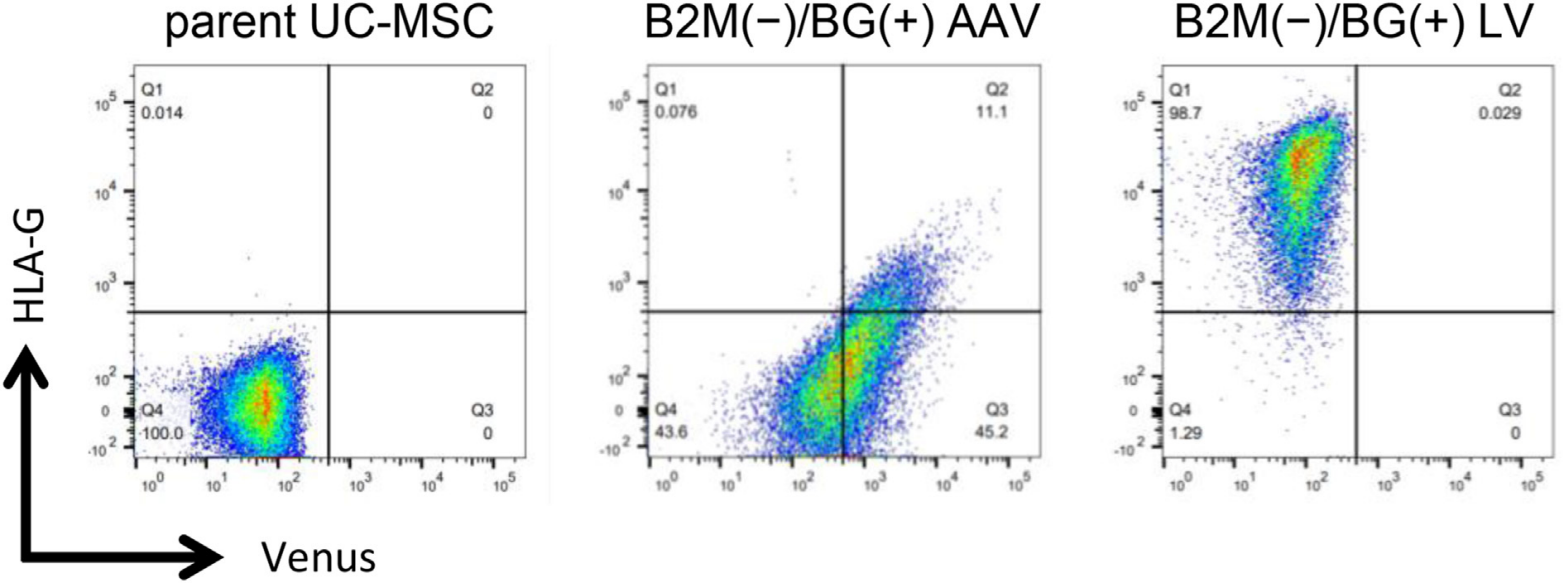
**B2M(−)/BG(+) LV expressed more HLA-G protein on its cell surface than B2M(−)/BG(+) AAV.** FCM analysis was performed to each UC-MSCs.

The killing assay co-culturing UC-MSCs and KHYG-1, an NK cell line, was used to assess the ability of UC-MSCs to evade the cytotoxic NK attacks. The result with B2M(−) showed that almost all cells were killed. The susceptibility of B2M(−) to the attack by KHYG-1, an NK cell line, may be because cell surface HLA-class I molecules were eliminated owing to B2M gene disruption. However, B2M(−)/BG(+) LV had the highest survival rate among the UC-MSCs tested, and this result indicates that B2M(−)/BG(+) LV escaped the cytotoxicity significantly more effectively than B2M(−) in the KHYG-1 co-culture system. The survival rate of B2M(−)/BG(+) AAV was also significantly higher than that of B2M(−), but was significantly lower than that of B2M(−)/BG(+) LV. Differences in the number of gene copies introduced may explain why B2M(−)/BG(+) AAV and B2M(−)/BG(+) LV differed in the survival rate, while both cell lines expressed the B2M-HLA-G fusion protein and HLA-G on the cell surface could serve a role in the immune escape from KHYG-1. A B2M(−)/BG(+) LV cell had multiple copies of the fusion gene at various positions in the genome as a result of random integration by lentivirus. In contrast, a B2M(−)/BG(+) AAV cell underwent the target region-specific sequence insertion and thus had only one or two copies in the genome theoretically (as long as off-target effect is negligible). Owing to the presence of such a difference in the number of copies in the genome, B2M(−)/BG(+) LV expressed more HLA-G on the cell surface than B2M(−)/BG(+) AAV; thus, it could more stably escape the attacks by KHYG-1. In fact, a comparison of HLA-G fluorescence intensities measured with FCM between B2M(−)/BG(+) AAV and B2M(−)/BG(+) LV confirmed that B2M(−)/BG(+) LV had a higher intensity (Fig. 5). Thus, using lentivirus has the advantage that the number of integrated gene copies become large and the expression level is also high. However, using lentivirus may induce insertional mutagenesis as a result of random integration depending on the inserted site. Since the target site can be specified in genome editing, it is more favorable in terms of safety. Taken together, the results suggest that UC-MSCs without HLA-A, B, and C can escape attacks by NK cells equally or more effectively compared to parent UC-MSC by the action of the B2M-HAL-G fusion gene product. Furthermore, the killing assay co-culturing UC-MSCs with PBMCs from healthy donors showed that B2M(−)/BG(+) AAV had the highest immune evasion ability. This result is consistent with our initial prediction. Meanwhile, the dead cell rates of B2M(−)/BG(+) LV remained high over time. These results suggest that B2M(−)/BG(+) LV could not escape the cytotoxicity of PBMCs or particularly CD8-positive T cells, while B2M(−)/BG(+) LV acquired the ability to escape from NK cells as expected. The most predictable reason is the influence of random integration by lentivirus, but exact reason could not be identified in this study.

Moreover, the survival rate of B2M(−)/BG(+) AAV co-cultured with PBMCs was significantly higher than that of parent UC-MSC co-cultured identically, particularly after 70 h. The enhancement of the immune escape ability of MSCs can be considered as a beneficial outcome of the AAV-mediated B2M-HLA-G gene introduction. When the survival rates of B2M(−)/BG(+) AAV were compared with those of B2M(−)/BG(+) LV over time, the survival rates of B2M(−)/BG(+) AAV were significantly higher, and the difference increased with the culture duration. These data show the possibility that the target gene-specific HLA-G knock-in can impart the sufficient immune evasion ability to the cells, without the requirement for HLA-G overexpression, and is more effective particularly for applications requiring long-term persistence. In this study, we did not monitor effects on cells co-cultured for more than 70 h. Moving forward, the ability to escape immune responses for a longer term may need to be tested.

Similar to the killing assay, the luciferase reporter assay also confirmed the tendency of the ability of UC-MSCs to evade the attacks by PBMCs. However, the survival rate of B2M(−)/BG(+) AAV tended to be lower than that of parent UC-MSC when the E/T ratio was higher. The reason underlying this tendency should be addressed in future analyses.

## 5 Conclusions

In this study, a gene introduction method combining the CRISPR/Cas9 system and AAV was used to create UC-MSCs effectively knocked out B2M gene and knocked in B2M-HLA-G fusion gene at the target site simultaneously. These UC-MSCs were capable of evading allogeneic immune responses of NK cells and T cells and surviving for a long period of time, while preserving characteristics as MSCs, such as cell surface marker profile and differentiation potentials. As UC-MSCs engineered in this manner are less susceptible to elimination by the host, they are expected to persist after transplantation, thereby exerting therapeutic effects for a long-term. Furthermore, these UC-MSCs are considered to be advantageous for use in regenerative medicine. In the future, it may be necessary to investigate short- and long-term *in vivo* cell dynamics and effects on immune responses toward application of the gene-edited UC-MSCs produced in this study for treatment of GVHD and other diseases.

## Authors' contributions

SM and RN performed the research, analyzed, and interpreted the data, and wrote the manuscript. TNI provided research samples. TNI and TO assisted the research. MF designed the research and edited the manuscript. AT supervised the projects

## Declaration of competing interest

RN is employed by Daiwa Pharmaceutical. SM is employed by Keijinkai Medical Corporation. AT received a research grant from Daiwa Pharmaceutical. TN-I, TO, and MF has no conflicts of interest.
