# Supplementary material for: CRISPR/Cas9 and AAV mediated insertion of β2 microglobulin-HLA-G fusion gene protects mesenchymal stromal cells from allogeneic rejection and potentiates the use for off-the-shelf cell therapy
Source: Regen Ther. 2022 Oct 14;21:442–52. doi: 10.1016/j.reth.2022.09.009 (PMC9582586; doi:10.1016/j.reth.2022.09.009)
Supplement: Multimedia component 1 [file mmc1.docx]

**CRISPR/Cas9 and AAV mediated insertion of β2 microglobulin-HLA-G fusion gene protects mesenchymal stromal cells from allogeneic rejection and potentiates the use for off-the-shelf cell therapy**

Sohsuke Meshitsuka, Ryo Ninomiya, Tokiko Nagamura-Inoue, Takashi Okada,

Muneyoshi Futami & Arinobu Tojo

**Supplementary information**

Supplementary Table 1

Supplementary Table 2

**Supplementary Table 1. CRISPR/Cas9 target sequence and PAM sequence used in this study**

| Target gene of CRISPR/Cas9 | Target sequence/PAM sequence (5´ to 3´) |
| --- | --- |
| B2M#1 | GGCCACGGAGCGAGACATCT/CGG |

**Supplementary Table 2. Antibodies for FCM used in this study**

| Antigen recognized | Label | Clone name | Manufacturer |
| --- | --- | --- | --- |
| CD90 | FITC | 5E10 | BioLegend |
| CD90 | BV421 | 5E10 | BioLegend |
| CD73 | APC-Cy7 | AD2 | BioLegend |
| CD105 | PE-Cy7 | 43A3 | BioLegend |
| CD34 | PE | 581 | BioLegend |
| CD11b | PE | ICRF44 | BioLegend |
| CD19 | PE | HIB19 | BioLegend |
| CD45 | PE | HI30 | BioLegend |
| HLA-DR | PE | L243 | BioLegend |
| CD4 | PE-Cy7 | A161A1 | BioLegend |
| CD8a | PE | HIT8a | BioLegend |
| HLA-G1, HLA-G5 | FITC | MEM-G/9 | abnova |
| HLA-G | biotin | MEM-G/9 | invitrogen |
| B2M | biotin | 2M2 | BioLegend |
| HLA-ABC | FITC | B9.12.1 | Beckman Coulter |
